# Intraoral Schwannomas: Presentation of a series of 12 cases

**DOI:** 10.4317/jced.51176

**Published:** 2013-10-01

**Authors:** José M. Sanchis, Claudia M. Navarro, José V. Bagán, Miriam A. Onofre, Judith Murillo, Cleverton R. De-Andrade, Jose M. Díaz, Valfrido A. Pereira-Filho

**Affiliations:** 1 Service of Stomatology and Maxillofacial Surgery. Hospital General Universitario de Valencia. Valencia, Spain; 2 Department of Diagnosis and Oral Surgey Dental School-UNESP, Araraquara, SP, Brazil

## Abstract

Introduction: Schwannomas are benign and not very frequent tumors of the peripheral nerves, derived from the nerve supporting Schwann cells. Study Design: Data were collected on the clinical manifestations (sex, age), location, size and symptonts of the lesions as well as the evolution time and the initial (presumption) diagnosis. Results: Twelve patients were documented, with a mean age of 29,5 ± 12,1 years (range 16-50) and a balanced gender distribution. The mean duration of the lesions was 42,17± 45,3 months. The lesion located in the floor of the mouth was the largest tumor, measuring about 4 cm in maximum diameter, while the average size of the 12 schwannomas was 2.04± 1.1 cm. Conclusion: We present 12 oral schwannomas diagnosed and treated over a period of 10 years.

** Key words:**Schwannomas, oral benign tumor, neurilemmoma.

## Introduction

Schwannomas or neurilemmomas are benign and not very frequent tumors of the peripheral nerves, derived from the nerve supporting Schwann cells. The first description of this type of tumor was made by Verocay in 1910, and although these are infrequent lesions, between 25-48% are located in the cervicofacial territory (1,2). The underlying etiology is not known. Schwannomas generally manifest as asymptomatic solitary nodules, with no gender predilection, and with a typical patient age at onset of between 20-50 years – though recently the presence of lingual schwannomas has been described in children between 10-13 years of age (3-5).

Schwannomas can be divided into central or intraosseous and peripheral lesions. In turn, a variant known as ancient schwannoma contains degenerative phenomena such as cystic cavities, hemorrhage, hyalinization or calcifications (6,7). A variant characterized by a nodular growth pattern (plexiform intraosseous schwannoma) has also been described (8).

Intraoral peripheral schwannomas are fundamentally located in the tongue, followed by the palate, floor of the mouth, cheek mucosa and gums (4,9-11). In turn, intraosseous schwannomas are most often located in the mandible (12-14), though there have been descriptions of cases in the intramasseteric region (15), zygomatic arch (16) or parotid zone, affecting the facial nerve (17,18).

Schwannomas are slow-growing and asymptomatic, and the differential diagnosis must be established with other clinically similar tumors such as neuromas, neurofibromas, granular cell myoblastoma, neuroepitheliomas, fibromas or adenomas (9). The tumor most often manifests as a painless mass or swelling, and usually evolves over a long period of time (even several years) (8,9,19).

Imaging techniques such as computed tomography (CT) or magnetic resonance imaging (MRI) can offer valuable information, showing a homogeneous, very well delimited and solid lesion. However, the definitive diagnosis requires biopsy-removal of the lesion (20) and histological study – revealing an encapsulated lesion with two characteristic tissue patterns referred to as Antoni A and Antoni B (8,21). Immunohistochemically, the lesion proves positive for protein S-100 (a specific neural tissue marker), vimentin, or other more specific markers such as NSE (neuron-specific enolase) (22).

Treatment consists of complete removal of the encapsulated lesion. The causal nerve is rarely identified, and relapses or neurosensory defects after the operation are exceptional (19,15,23,24). Likewise no neurological sequelae have been reported in those cases arising from the mental nerve (25), mylohyoid nerve (10) or sublin-gual gland (22).

The present study describes a series of 8 cases of intraoral schwannoma diagnosed and treated in the Department of Stomatology and Maxillofacial Surgery (Valencia University General Hospital, Valencia, Spain) during the period 2002-2011 (cases 1-8) and 4 cases reported by the Department of Diagnosis and Oral Surgery (Dental School UNESP, Araraquara, SP, Brazil), diagnosed and treated during the same period 2003-11 (cases 9-12).

## Clinical Cases

**Figure 1:**
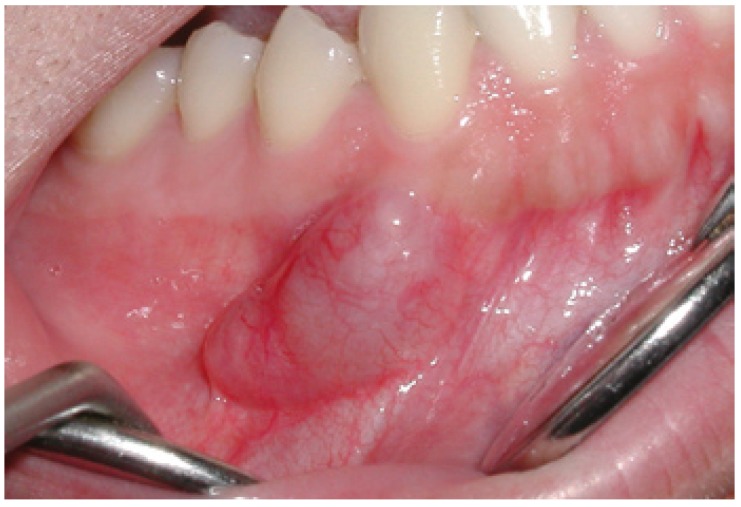
Case three. Clinical view.

**Figure 2:**
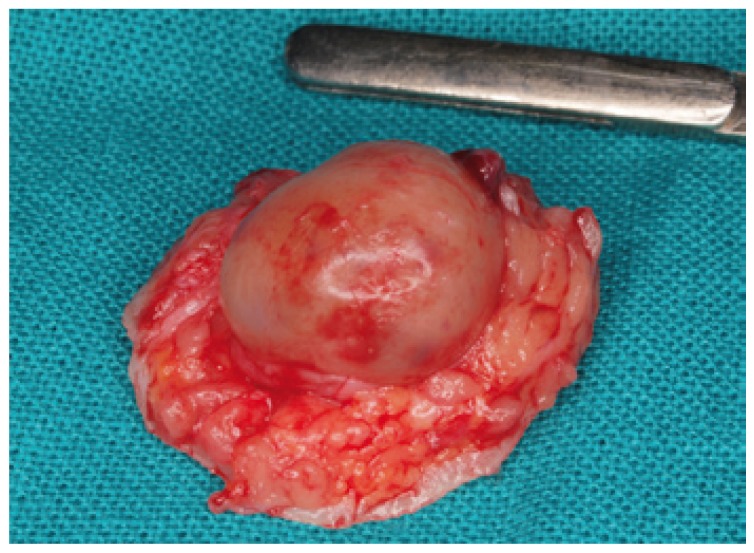
Case six. Resection piece.

Twelve patients were documented, with a mean age of 29,5 ± 12,1 years (range 16-50) and a balanced gender distribution ( Table 1). The mean duration of the lesions was 42,17± 45,3 months. The lesion located in the floor of the mouth was the largest tumor, measuring about 4 cm in maximum diameter, while the average size of the 12 schwannomas was 2.04± 1.1 cm. In case 2, corresponding to the lesion in the floor of the mouth, was some discomfort in speech, chewing and swallowing reported, as a consequence of the size of the tumor. (Figs. 1,2).

**Table 1:**
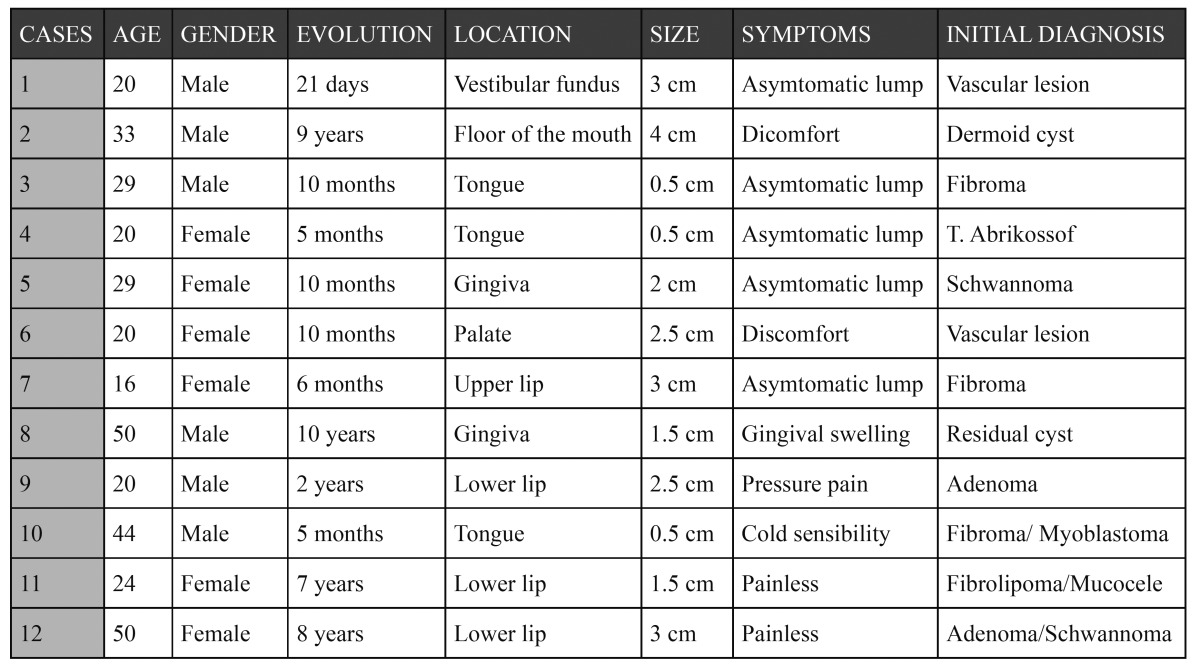
Clinical characteristics of the 12 patients with oral Schwannoma.

Magnetic resonance imaging was performed in three cases, and in two of them (corresponding to the largest lesions) fine needle aspiration biopsy (FNAB) and prior biopsy were carried out. The two largest lesions (floor of the mouth and palate) were completely removed under general anesthesia, while in the remaining 10 cases the tumor was removed under local anesthesia. There were no postoperative complications or relapses in any of the cases.

A histopathological diagnosis of schwannoma was established in all cases, and the immunohistochemical study revealed positivity for protein S-100, with a Ki-67 cell proliferation index of 4% and 5% in cases 1 and 4, respectively.

## Discussion

Schwannomas located in the maxillofacial territory account for 30-40% of all such tumors (1,2). Leu et al. (26) described 52 cases located in the head and neck and documented over a period of 8 years – only 7 of the lesions being located in the oral cavity. Lacosta and Zabaleta (24) in turn described 7 cases in the maxillofacial region recorded during a period of 19 years in a hospital in Logroño (Spain) (3 in the tongue and one in the cheek mucosa). Likewise in Spain and covering a period of 17 years, a total of 9 intraoral schwannomas (21) were documented in two hospitals in Madrid and Bilbao. In our series, a total of 8 intraoral schwannomas were diagnosed and treated during a period of 9 years. The estimated frequency of such lesions in the oral cavity is therefore low. Do Nascimento et al. (2) described these tumors as representing 0.04% of all intraoral lesions, since they documented four cases of schwannoma among 9000 biopsies in a Pathology Department in Brazil over a period of 38 years. Salla et al. (1) in turn described four cases (0.02%) among all the intraoral biopsies performed in another Brazilian Pathology Department over a period of 40 years. In our serie, the 4 brazilian cases were collected among 1331 performed biopsies during a period of 8 years.

Although schwannoma appears to show a slight male predilection (26), some authors have published series involving only women (1). In any case, the gender differences are not significant (2,21-24), as evidenced by our own balanced distribution (6 males and 6 females). The age at presentation of the disease varies considerably, though with a predominance in early ages. The mean age in the main series ranges from 23.7 years (1) to 38.2 years (24), while in our series the mean age was 27.1 years. When the lesions are located in the tongue, they can also be identified in children between 10-13 years of age (3-5). Cases identified at 70 (4,14) or 80 years of age are exceptional (7).

Clinically, schwannomas produce few symptoms and are usually recognized from the presence of a slow-growing mass. They only rarely manifest as an ulcerated or infected lesion (27). As in case 2 of our series (located in the floor of the mouth), this characteristically slow growth and lack of symptoms can cause the lesions to be first diagnosed after more than 5 years (9,19). The most frequently described symptom of intraoral schwannomas is swelling and pain (23), though none of our patients experienced pain. Nevertheless, most authors describe discomfort associated to the swelling in the palate (14,20) or tongue (3-5,9). No sensory disturbances have been described, and the references to mandibular intraosseous schwannomas (6,8,13,28) describe slightly painful swelling, but without paresthesias.

A tentative clinical diagnosis of schwannoma is difficult to establish, and identification of the lesion sometimes comes as a surprise (29). Wang et al. (30) reviewed the tentative diagnosis of 26 biopsied schwannomas, and found 8 of the lesions to be intraoral, with a wrong diagnosis of tongue fibroma (4 cases), dermoid cyst (2 cases) and salivary gland tumor (2 cases). Our initial diagnosis proved correct in two cases, while the tentative diagnosis was fibroma in 4 cases, vascular lesion in two cases, Abrikossof tumor, dermoid cyst and adenoma in one case; and residual cyst in another case. In their series of four cases, Do Nascimento et al. (2) acknowledged that while one lesion was clinically diagnosed as corresponding to neurofibroma, the clinical diagnosis was not correct in the other three cases. Lopez and Ballestin (21) clinically diagnosed one case of neurinoma, while the remaining 8 cases in their series received a wrong initial diagnosis.

Among the complementary diagnostic techniques, mention must be made of the panoramic X-ray study to discard associated dental lesions or the involvement of bone in proximity to the lesion. A routine panoramic X-ray study was carried out in all of our patients. However, the mucous and exophytic nature of schwannomas requires the use of MRI, which is the diagnostic technique offering the largest body of information in such cases. MRI was used in three of our patients. A biopsy may be indicated in very large lesions or cases characterized by important diagnostic uncertainty. In our patients with tumors in the floor of the mouth and in the palate, biopsies were obtained to confirm the diagnosis.

**Figure 3:**
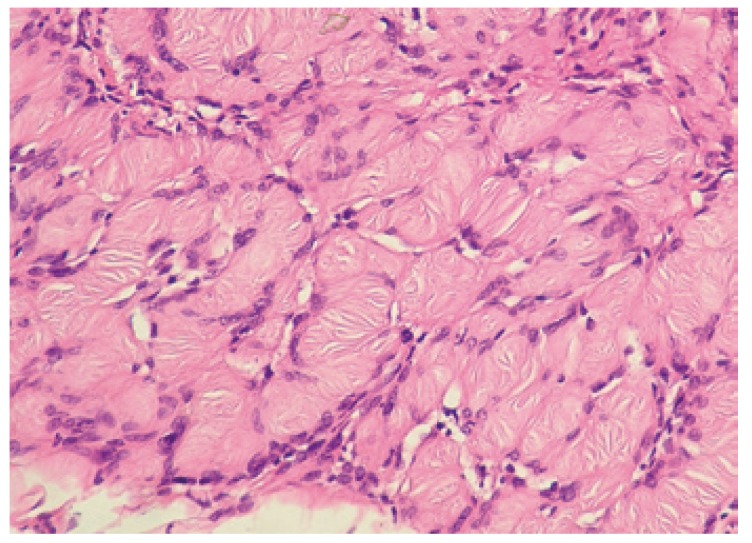
Histopathological findings. Case eleven: Proliferated spindle-shaped tumor cells with palisading patterns in the cell-rich area..

The definitive diagnosis of schwannoma is established by the histopathological study of the lesion, with the presence of the two typical tissue patterns referred to as Antoni A and Antoni B. The first of these patterns is characterized by the presence of cells with elongated or fusiform nuclei adopting a palisade distribution (Fig. 3). In contrast, the Antoni B pattern corresponds to cells and fibers lacking a specific orientation, with increased separation among the cells and a disorderly distribution with the presence of interstitial edema and microcysts (21,24). Another typical finding corresponds to the so-called Verocay bodies, which are small hyaline structures. Immunohistochemically, protein S-100 (a specific neural tissue marker) is seen to be positive. All of our cases were diagnosed on the basis of the typical cell structure, with the immunohistochemical confirmation of protein S-100 positivity.

The treatment of schwannomas consists of surgical removal of the tumor, with preservation of the neighboring structures. Relapses are very rare. These lesions usually cause no sensory defects, while Lacosta et al. (24) in their review indicated that while rare, schwannoma malignization may occur. None of our patients suffered relapses or neurological sequelae.

## References

[1] Salla JT, Rodrigues ACB, Gonçalves B, Ferreira MC, Alves R (2009). Retrospective analysis of oral peripheral nerve sheath tumors in Brazilians. Braz Oral Res.

[2] Do Nascimento GJF, De Albuquerque D, Cavalcanti H, Lopes AL, De Souza LB (2011). A 38-year review of oral schwannomas and neurofibromas in a Brazilian population: clinical, histopathological and immunohistochemical study. Clin Oral Invest.

[3] Luksic I, Müller D, Virag M, Manojlovic S, Ostoviv KT (2011). Schwannoma of the tongue in a child. J Craniomaxillofac Surg.

[4] Karaca CT, Habesoglu TE, Naibouglu B, Habesoglu M, Oysu C, Egeli E (2010). Schwannoma of tongue in a child. Am J Otolaryngol.

[5] Naidu GS, Sinha SM (2010). Schwannoma of the tongue: an unusual presentation in a child. Indian J Dent Res.

[6] Jahanshahi G, Haghighat A, Azmoodeh F (2011). Intraosseus neurilemmoma of the mandible: report of a rare ancient type. Dent Res J (Isfahan).

[7] Humber C, Copete MA, Hohn FI (2011). Anciant schwannoma of upper lip: report with distinct histologic features and review of the literature. J Oral Maxillofac Surg.

[8] Vera-Sempere F, Vera-Sirera B (2010). Intraosseous plexiform schwannoma of the mandible: immunohistochemical differential diagnosis. J Craniofac Surg.

[9] Jeffcoat BT, Pitman KT, Brown AS, Baliga M (2010). Schwannoma of the oral tongue. Laryngoscope.

[10] Pattani KM, Dowden K, Nathan CO (2010). A unique case of a sublingual-space schwannoma arising from the mylohyoid nerve. Ear Nose Throat J.

[11] Isildak H, Yilmaz M, Ibrahimov M, Aslan M, Karaman E, Enver O (2010). Schwannoma of the hard palate. J Craniofac Surg.

[12] Sun Z, Sun L, Li T, Ma X, Zhang Z (2011). Intraosseous trigeminal Schwannoma of mandible with intracranial extension. J Laringol.

[13] Patil K, Mahima VG, Srikanth HS, Saikrishna D (2009). Central schwannoma of mandible. J Oral Maxillofac Pathol.

[14] Shetty SR, Mishra C, Shetty P, Kaur A, Babu S (2012). Palatal schwannoma in an elderly woman. Gerodontology.

[15] He Y, Fu HH, He J, Zhu HG, Zhang ZY (2010). Schwannoma arising from intramasseteric region. J Craniofac Surg.

[16] Shah A, Latoo S, Ahmad I, Malik AH, Singh AP, Hassan S (2011). Schwannoma causing resorption of zygomatic arch. J Oral Maxillofac Pathol.

[17] Quin MA, Song H, Zhang P, Hou R, Cheng X, Lein D (2010). Diagnosis and management of intraparotid facial nerve schwannoma. J Craniomaxillofac Surg.

[18] Zhong L, Wang L, Ji T, Yang W, Zhang C (2011). Management of facial nerve schwannoma in the accessory parotid region. J Oral Maxillofac Surg.

[19] Martins MD, Anunciato De Jesus L, Gernandes KP, Busadori SK, Taghloubi SA (2009). Intra oral schwannoma: case report and literature review. In J Dent Res.

[20] Lollar KW, Pollak N, Liess BD, Miick R, Zitsch RP (2010). Schwannoma of the hard palate. Am J Otolaryngol.

[21] Lopez JL, Ballestin C (1993). Intraoral schwannoma. A clinicopathological and immunohistochemical study of nine cases. Arch Anat Cytol Pathol.

[22] Okada H, Tanaka S, Tajima H, Akimoto Y, Kaneda T, Yamamoto H (2011). Schwannoma arising from the sublingual gland. Ann Diagn Pathol.

[23] De Andrade Santos P, Souza V, Pereira L, de Almeida R, de Souza LB (2010). Clinicopathologic analysis of 7 cases of oral schwannoma and review of the literature. Ann Diagnostic Pathol.

[24] Lacosta J, Zabaleta M (1999). Extracranial schwannomas. Report of seven cases. Acta Otorrinolaringol Esp.

[25] Subhashraj K, Balanand S, Pajaniammalle S (2009). Ancient schwannoma arising from mental nerve. A case report and review. Med Oral Patol Oral Cir Bucal.

[26] Leu YS, Chang KC (2002). Extracranial head and neck schwannoma: a review of 8 years experience. Acta Otolaringol.

[27] Khonsari RH, Perrin JP, Bouguilla J, Billet J, Corre P (2009). Acute presentation in oral schwannoma. Rev Stomatol Chir Maxillofac.

[28] Shetty SR, Rao PK, Chatra L, Shenai P (2011). A case of massive mandibular schwannoma. J Neurosciences in Rural Practice.

[29] Cardoso CL, Tolentino Ede S, Capelozza AL, Consolaro A (2010). Schwannoma in the lower lip mucosa: unexpected diagnosis. Quintessence Int.

[30] Wang XX, Zhang J, Wei FC, Zhao ZQ (2004). Analysis of preoperative misdiagnosis causes in 26 cases of neurilemmoma. Shanghai Kou Quiang YI Xue.

